# A proof-of concept randomized controlled trial to show that the antidepressant effect of psilocybin does not require a psychedelic experience: study protocol

**DOI:** 10.1192/j.eurpsy.2023.1175

**Published:** 2023-07-19

**Authors:** M. I. Husain, D. M. Blumberger, D. J. Castle, S. M. Kloiber, A. Ortiz, J. D. Rosenblat, B. H. Mulsant

**Affiliations:** 1 Campbell Family Mental Health Research Institute, Centre for Addiction and Mental Health; 2 Psychiatry, University of Toronto; 3 Psychiatry, University Health Network, Toronto, Canada

## Abstract

**Introduction:**

During the last decade there has been a resurgence of interest on the use of psychedelics as novel treatments for mental disorders, including treatment-resistant depression (TRD). Psilocybin, the chemical component of “magic mushrooms”, has been administered with psychotherapy in randomized clinical trials (RCTs) showing large and sustained antidepressant effects. As the use of psilocybin expands, it is becoming more important to understand whether psilocybin’s psychedelic effects are required for psilocybin’s antidepressant effects. Psilocybin’s psychedelic effects are known to be dependent on serotonin 2A receptor (5-HT2AR) activation. Given the safety concerns associated with psilocybin’s psychedelic effects, all studies have used it in conjunction with at least 12 hours of intensive psychotherapy. This makes psilocybin-assisted psychotherapy (PAP) highly resource intensive and impedes scalability given limited resources and access to trained therapists in most jurisdictions. Studies in healthy volunteers have shown that psilocybin’s psychedelic effects are blocked by 5-HT2AR antagonists like risperidone and ketanserin. In a pre-clinical study using a mouse model of depression, administration of ketanserin followed by psilocybin had the same antidepressant effect as psilocybin alone. We propose to conduct the first study to test in humans whether the antidepressant effects of psilocybin are attenuated by 5-HT2AR blockade from risperidone.

**Objectives:**

Aim 1: To evaluate the feasibility and tolerability of administering psilocybin with risperidone in adults with TRD by evaluating recruitment, retention, tolerability, and safety.

Aim 2: To evaluate psychedelic effects (measured with the 5-Dimensional Altered States of Consciousness Rating Scale) in the three groups.

Aim 3: To evaluate antidepressant effects (measured with the Montgomery Asberg Depression Rating Scale; MADRS) in the three groups. .

**Methods:**

A three-arm, 4-week, double blind, proof-of-concept RCT for patients with a DSM-5 major depressive episode that has failed to respond to at least two adequate trials of antidepressants. Participants will be randomized to: 1) psilocybin 25 mg plus risperidone 1 mg; 2) psilocybin 25 mg plus placebo; 3) placebo plus risperidone 1 mg. All participants will receive 12 hours of manualized psychotherapy.

**Results:**

Ethics approval for the proposed study has been obtained. We will present preliminary feasibility data at the meeting in March.

**Conclusions:**

If the study demonstrates that psilocybin’s psychedelic effects are not necessary for psilocybin’s antidepressant effects, the combination of psilocybin and a 5-HT2AR antagonist, such as risperidone, could increase acceptability and access to the use of psilocybin to treat MDD and related conditions.

**Disclosure of Interest:**

None Declared

